# Unadjuvanted antenatal pertussis OMV vaccination drives enhanced and durable humoral immunity in offspring

**DOI:** 10.3389/fimmu.2026.1883311

**Published:** 2026-08-04

**Authors:** Oriana López, María Emilia Gaillard, Pablo Martin Aispuro, Lucia Locati, Bernarda Pschunder, María Eugenia Zurita, Camila Escudero, Daniela Bottero, Daniela Hozbor

**Affiliations:** Laboratorio VacSal, Instituto de Biotecnología y Biología Molecular (IBBM), Facultad de Ciencias Exactas, Universidad Nacional de La Plata, CCT-CONICET -La Plata, La Plata, Argentina

**Keywords:** antenatal, *Bordetella pertussis*, immunization, offspring protection, OMVs, pertussis

## Abstract

**Background:**

Antenatal-immunization is the primary strategy to protect neonates from pertussis. However, the global shift to acellular vaccines (aP) has raised concerns regarding the duration of transferred immunity, largely due to the Th2-skewed profile they induce. This study evaluates the immunogenicity and protective efficacy transferred to offspring by a novel, unadjuvanted *Bordetella pertussis* Outer Membrane Vesicle-based vaccine (OMVPreg), which induces a mixed Th1/Th2/Th17 response, compared to the commercial aP vaccine (aPPreg).

**Methods:**

Female mice received a primary two-dose series of aP followed by an antenatal booster of either OMVPreg or aPPreg. Offspring from successive litters were intranasally challenged with *B. pertussis* at neonatal (day7), infant (day21), and adult (day60) stages. We first characterized the maternal-offspring humoral response and subsequently quantified offspring protection through pulmonary bacterial clearance (log10CFU), correlating these outcomes with antibody kinetics and cellular immune profiles measured by ELISA.

**Results:**

Both vaccines were immunogenic in dams, albeit with distinct profiles. The primary two-dose aP schedule, as well as the aPPreg booster, elicited a predominantly IgG1 isotype response (Th2-skewed), whereas OMVPreg induced a mixed IgG1/IgG2a/IgG3 response. This balanced isotype profile was achieved despite prior aP priming, indicating that the OMV booster effectively redirected maternal immunity. The isotype polarization was faithfully transferred to offspring, and IgG-levels in OMVPreg pups remained stable across successive litters, unlike aPPreg pups which showed a significant decline. Upon challenge, OMVPreg offspring exhibited an age-dependent mixed cellular profile: neonates produced higher IFN-γ and IL-22, infants higher IL-17, and adults maintained higher IFN-γ compared to aPPreg counterparts (offspring born shortly after antenatal boost), confirming that OMVs provide essential Th1 signals deficient in aP-boosted offspring. Regarding protection, both vaccines provided equivalent bacterial clearance in early litters (within 12 weeks post-booster). However, in adult offspring from late-born litters (>14weeks), aPPreg-mediated protection waned (4.6 log_10_ CFU), whereas OMVPreg offspring maintained superior clearance (3.6 log_10_ CFU; p<0.05).

**Conclusion:**

Unadjuvanted OMVs effectively modulate the offspring’s immune environment, bridging the vulnerability gap left by acellular vaccines. Notably, this was achieved despite prior aP priming, demonstrating that the OMV booster redirects maternal immunity toward a balanced isotype profile and a mixed Th1/Th17-antibody isotype signature response. This translated into long-term protection against severe pulmonary disease in offspring, even as passive maternal antibodies waned.

## Introduction

Pertussis, commonly known as whooping cough, is a highly contagious respiratory disease caused primarily by the Gram-negative bacterium *Bordetella pertussis*. Despite long-standing vaccination programs, few decades ago the disease has resurged globally, with infants under six months of age facing the highest risk of severe complications and mortality (1–5)⁠. To close the immunity gap between birth and the first infant pertussis dose, antenatal immunization has emerged as a key strategy (6, 7)⁠. Since 2011, an increasing number of countries have recommended vaccination against pertussis during pregnancy, with Argentina and the United Kingdom being global pioneers after implementing national programs in 2012 (8–11)⁠. A large body of evidence supports that this strategy is safe for pregnant women, developing fetuses, and infants (11–13)⁠. Effectiveness studies have shown that antenatal pertussis vaccination (acellular pertussis vaccine, aP) prevents 69–93% of laboratory-confirmed pertussis infections, 91–94% of hospitalizations, and up to 95% of pertussis-related deaths in infants under 2–3 months of age (10, 13, 14)⁠. Given these substantial benefits, the universalization of antenatal vaccination strategies remains a key public health goal.

Despite this clear success, current aP vaccines have inherent limitations that warrant further improvement. Acellular vaccines containing aluminum adjuvants induce a predominantly humoral and Th2-skewed response (15–18). Pregnancy is generally considered a Th2-polarized environment (19), which may influence the response to vaccines administered during gestation⁠. Thus, aP-induced immunity is short-lived, which is why vaccination is recommended during every pregnancy (13)⁠. These repeated immunizations lead to high levels of maternal antibodies that can interfere with the infant’s own immune response to active vaccination, a phenomenon known as blunting effect driven by mechanisms such as epitope masking and engagement of the inhibitory FcγRIIB receptor (CD32) on neonatal B cells (20)⁠. Thus, while the current strategy is highly effective, there is a need to improve the aP vaccines by developing formulations that induce a more balanced and durable immune profile, potentially mitigating the need for revaccination in every pregnancy and reducing blunting.

Following the COVID-19 pandemic, the epidemiological landscape has shifted significantly, with a notable rise in infections among adolescents and young adults (21, 22)⁠. COVID-19 mitigation measures also led to a significant decrease in *B. pertussis* circulation during 2020–2021, which may have negatively affected natural boosting and population immunity. This epidemiological change does not imply that the most vulnerable, newborns and young infants, are no longer at risk. On the contrary, the increased circulation of *B. pertussis* in adolescents and adults, who often serve as asymptomatic or pauci-symptomatic reservoirs, heightens the risk of transmission to unprotected infants (23)⁠. Furthermore, during and after the pandemic, there has been a concerning decline in vaccination coverage in many regions, driven in part by an unprecedented erosion of vaccine confidence (24–27)⁠. Thus, while efforts to optimize vaccination coverage and maximize the use of existing vaccines must continue, these strategies alone may not fully address the immunological shortcomings of current aP vaccines (3, 18, 28, 29)⁠. New vaccine candidates designed to overcome the intrinsic limitations of current pertussis vaccines are urgently needed (30–32)⁠. In this context, our group has proposed outer membrane vesicles (OMVs) derived from *B. pertussis* as a next-generation vaccine prototype that directly addresses these limitations (33–39)⁠. OMVs are nano-sized structure naturally shed by *B. pertussis* that contain a complex array of membrane-associated antigens in their native conformation, along with endogenous adjuvants such as lipopolysaccharides and other PAMPs (40–42)⁠. Unlike aP vaccines, OMVs can induce lung-resident memory CD4+ T cells and balanced Th1/Th17/Th2 immune profile without the addition of exogenous adjuvants (36, 39)⁠. This balance is particularly relevant in the context of antenatal immunization, where the goal is to transfer the immune profile required for pertussis protection to the offspring. Furthermore, OMVs retain the immunogenicity of whole cell pertussis vaccines (wP) while avoiding the safety concerns that contraindicate wP use in people over 7 years of age.

In this study, we compared the efficacy of an unadjuvanted OMV-based booster (OMVPreg) versus a commercial aP booster (aPPreg) administered during pregnancy in a murine model. Both groups received an identical aP priming series prior to pregnancy, such that the only difference between experimental groups was the antenatal booster administered. We specifically evaluated the immunogenicity in dams, the transfer and persistence of maternal antibodies in offspring across successive litters, and the protective efficacy against sublethal respiratory challenge at different developmental stages (neonatal, infant, and adult). Our central hypothesis was that, unlike aP vaccines, an OMV-based antenatal booster would induce a more balanced and durable immune profile conferring sustained protection even as passive maternal antibodies wane.

## Materials and methods

### Mice

BALB/c mice (3–4 weeks old), obtained from the Faculty of Veterinary Sciences, La Plata, Argentina, were kept in ventilated cages and housed under standardized conditions with regulated daylight, humidity, and temperature. The animals received food and water ad libitum. Day 1 of gestation was determined when a vaginal plug was observed. Breeding cages were checked daily for new births, and pups were kept with their mothers until weaning at the age of 3 weeks. The animal experiments were authorized by the Ethical Committee for Animal Experiments of the Faculty of Science at La Plata National University (approval number 004 - 06-15, 003 - 06–15 extended its validity until August 10, 2027).

### *B. pertussis* strain and growth conditions

*B. pertussis* Tohama phase I strain CIP 8132-gentamicin resistant was used throughout this study as the strain for challenge in the murine model of protection. Bacteria were grown in Bordet–Gengou agar supplemented with 10% (v/v) defibrinated sheep blood (BG-blood agar) for 72h at 36.5 °C. Isolated colonies were replated in the same medium for 24h and then resuspended in phosphate-buffered saline (PBS: 123 mM NaCl, 22.2 mM Na_2_HPO_4_, 5.6 mM KH_2_PO_4_ in MilliQ^®^ nanopure water; pH 7.4). The optical density at 650 nm was measured and serial 10-fold dilutions plated onto BG-blood agar to determine the number of bacteria in the challenge inoculum.

### Outer membrane vesicle isolation

OMVs were isolated from *B. pertussis* grown in Stainer–Scholte liquid medium (43)⁠ during the decelerating growth phase as previously described (33)⁠. Briefly, bacterial obtained after centrifugation (8,000xg 30 minutes at 4 °C) was resuspended in 20 mM Tris–HCl, 2 mM EDTA, pH 8.5 and sonicated in cold water for 20 min. The free bacterial supernatant obtained by centrifugation was ultracentrifugated at 100,000×g for 2 h at 4 °C. OMVs were resuspended in 20 mM Tris buffer, pH 7.6. The obtained samples were negatively stained for electronic microscope examination and characterized by SDS-PAGE and immunoblotting to confirm the presence of major immunogenic proteins (35, 39)⁠. The PTx content in the OMV preparation was quantified by dot-blot analysis using purified His-tagged PTxA as a standard, with detection using an anti-His antibody, and found to be approximately 90 ng per 6 µg OMV dose. The methods for OMV characterization and antigen quantification were described in detail previously (39). Batch-to-batch consistency in PTx content has been routinely confirmed.

### Vaccines

Antenatal immunization was performed using either the 5-valent acellular pertussis vaccine Adacel^®^ (Sanofi) or an OMV-based vaccine. The composition per human dose of Adacel^®^ is: pertussis toxoid (2.5 µg), pertactin (3 µg), filamentous hemagglutinin (5 µg), fimbriae 2 and 3 (5 µg), tetanus toxoid (5 Lf), and diphtheria toxoid (2 Lf). The OMV vaccine was formulated with 6 µg of formol-detoxified OMV per dose, without adjuvant, supplemented with tetanus toxoid (0.5 Lf) and diphtheria toxoid (0.2 Lf). For all experiments, mice received a 1/10 human dose of the commercial vaccine or 6 µg of OMVs.

### Antenatal immunization

Female BALB/c mice (n=6) were intramuscularly vaccinated with 2 doses of aP at days 0 and 14 plus a booster dose of commercial aP vaccine (aPPreg) or Outer membrane vesicles derived from *B. pertussis* (OMVPreg) during pregnancy. After the second dose, female mice were housed with males within the same cage and daily checked for pregnancy, when mucosal vaginal plug was detected a third vaccine dose was applied. Mice couples stayed cohoused until the end of the experiment. Non-immunized mice were used as negative control of protection. From each dam, up to 7–8 litters were analyzed, with litter sizes ranging from 3 to 10 pups. Litters were classified into two groups: short-term (born between 3 and 12 weeks after the antenatal third dose) and long-term (born between 14 and 30 weeks after the antenatal third dose). Within each period, litters were statistically equivalent in terms of transferred immune response and acquired protective capacity. Offspring of both sexes were included in all of the analysis performed here, since in our extensive experience with this model, and consistent with the absence of reported sex-dependent differences in the literature, we have not observed sex-related differences. Pups from each period (minimum of 6 animals per litter) were randomly distributed to evaluate immune responses and protective capacity at different developmental stages: neonates, infants, and adults. Mean values from each litter, derived from different mothers, were compared in the analyses.

### Protection against colonization

To evaluate the protective efficacy induced by each vaccination strategy here tested, offspring from immunized dams were intranasally challenged with varying doses of *B. pertussis*. The challenge dose was adjusted according to the age group: neonates (7 days old), infant mice (21 days old), and adult mice (>2 months old) (see Figure Legends for details). Seven days post-challenge, mice were euthanized by cervical dislocation without prior sedation. This method is consistent with the AVMA Guidelines for the Euthanasia of Animals (2020) for small laboratory rodents when performed by trained personnel, and it was explicitly approved for this study by the Institutional Animal Care and Use Committee (CICUAL) of the Faculty of Exact Sciences, National University of La Plata (protocol approval No. 004-06-15, extended until 2027). Immediately after euthanasia, lungs and nose were aseptically harvested. The tissues were homogenized in PBS, and serial dilutions of the homogenates were plated onto BG-blood agar plates. Colony-forming units (CFUs) were counted after incubation at 37 °C for 3–4 days. At least three independent experiments were performed. The detection limit was 10 CFU per organ, with values below this threshold considered not detectable (as indicated by the dotted line in the corresponding figures).

### Enzyme-linked immunosorbent assay

As we previously described (42, 44, 45)⁠, plates (Nunc A/S, Roskilde, Denmark) were coated with heat killed *B. pertussis* lysate (HKBp) or purified pertussis toxin subunit A (PTxA) at 5 µg/ml or Tetanus toxoid (0.5 µg/ml) and Diphtheria toxoid (0.75 µg/ml) in 0.5 M carbonate buffer pH 9.5 with an overnight incubation at 4 °C. Blocked plates with 3% milk in PBS-Tween 0.05% (1h 37°C) were incubated with serially diluted samples of mouse serum (1h 37°C). Sera from dams and offspring from different periods were obtained by collecting blood from submandibular vein and leaving the blood samples to clot for 1h at 37°C followed by centrifuging for 10 min at 7,000xg. IgGs from individual serum bound to the plates were detected after a 1-h incubation with goat anti–mouse-IgG–linked horseradish peroxidase (1:8,000 BioRad, USA). For measuring IgG isotypes, detection of bound antibody was determined using HRP labeled subclass-specific anti-mouse IgG1 (1:3,000, Invitrogen USA), IgG2a (1:12,000 Invitrogen, USA) or IgG3 (1:1,500, Invitrogen USA). As substrate 1.0 mg/ml o-phenylendiamine (OPD, Bio Basic Canada Inc) in 0.1 M citrate-phosphate buffer, pH 5.0 containing 0.1% hydrogen peroxide was used. Optical densities (ODs) were measured with 800 TS Biotek microplate reader at 490 nm. From the experimental protocol performed in triplicate, one representative experiment is presented in the Results. The lower limit of detection for ELISA was determined as the mean OD of blank wells plus 2 standard deviations (corresponding to OD = 0.07).

### Ag-specific cytokine production by spleen cells

Spleens from untreated and immune mice were passed through a 40-mm cell strainer to obtain a single-cell suspension. Spleen cells were seeded in 48 well culture plates in a final volume of 500 µl/well RPMI 1640 with 10% fetal bovine serum, containing 100 IU/ml penicillin and 100 µg/ml streptomycin (35, 39)⁠. All cell samples were stimulated with heat killed *B. pertussis* suspension (10^6^ UFC/ml), or medium only. After 72 h of incubation (37°C and 5% CO_2_), IFN-γ, IL-17, and IL-22 concentrations were quantified in supernatants by ELISA.

### Statistical analysis

The normal distribution was assessed using the Shapiro-Wilk test (p > 0.05 was considered to indicate a normal distribution), and data were log-transformed when necessary to meet the assumptions of parametric tests. The data were evaluated statistically by two-way or one-way analysis of variance (ANOVA) followed by Tukey or Šidák for multiple comparisons (via the GraphPad Prism^®^ software). Differences were considered significant at a p <0.05.

## Results

### Maternal humoral immune response following antenatal boosting

**Figure 1:**
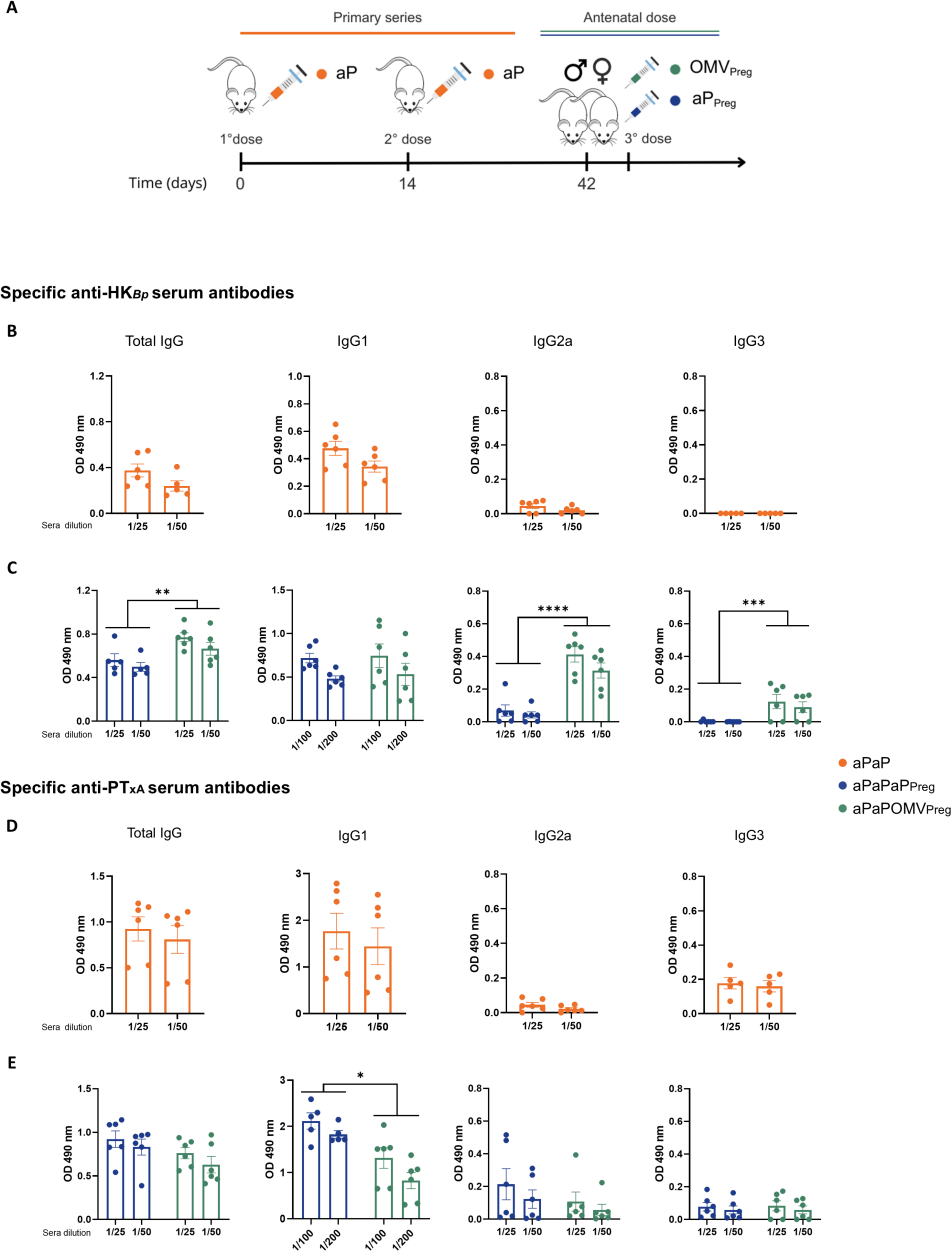
Specific *Bordetella pertussis* humoral immune response induced by antenatal immunization in a murine model. ​ Schematic representation of the experimental design is presented in **(A)** Female BALB/c mice of 3–4 weeks of age (n=6) received 2-doses of commercial acellular vaccine (1/10 human dose) as a primary series. Four weeks after the second dose, females were paired with males for mating. Pregnant mice were then boosted with an OMV-based vaccine (OMVPreg) or aP vaccine (aPPreg). Non-immunized females were included as a negative control (not shown). Specific *B. pertussis*-humoral immune response was evaluated after primary series **(B, D)** and booster dose **(C, E)**. Total IgG, IgG1, IgG2a and IgG3 levels against heat-killed *B. pertussis* lysate (HKBp, **B, C**) and pertussis toxin A (PTxA, **D, E**) were measured by ELISA at 28 days after the last dose. Optical density (OD) at 490 nm was determined for two serum dilutions. Statistical significance: *p<0.05, **p<0.01, ***p<0.001, ****p<0.0001 (two-way ANOVA, Sidak’s multiple comparisons)..

To evaluate the immunogenicity of two distinct antenatal booster strategies in aP-primed mice, we compared an unadjuvanted outer membrane vesicle (OMV) vaccine prototype (OMVPreg), which induces a mixed Th1/Th2/Th17 profile, and a commercial acellular vaccine (aPPreg), which elicits a predominantly Th2-biased response (**Figure 1**). The complete immunization schedule used is outlined in **Figure 1A**. Female mice received intramuscularly two doses of aP vaccine spaced 14 days apart. Fourteen days after the second dose (day 28), serum samples were collected to evaluate the humoral immune response established by the two-dose aP priming schedule (**Figures 1B, D**, orange symbols). At 28 days after the second dose, animals were then paired with males for mating. Upon detection of a vaginal plug, pregnant females received a single antenatal booster of either the unadjuvanted OMV protype vaccine or a commercial aP vaccine. Twenty-eight days after the antenatal boost, serum samples were again collected to evaluate the effect of the third dose. This design allowed us to determine what the two priming doses establish and, subsequently, what the antenatal booster adds. We first examined the humoral response against heat-killed *B. pertussis* (HKBp), which presents a complex array of bacterial antigens (**Figures 1B, C**). Total IgG levels (**Figure 1B**, left panel) showed that the aP 2-dose schedule exhibited significant antibody levels, establishing a solid baseline in comparison with a non-immunized group which served as a negative control. Both antenatal boosters further increased total IgG levels, with the OMVPreg group reaching the highest levels, significantly surpassing both the aP 2-dose priming schedule and the aPPreg group (p < 0.01). The isotype profile (**Figure 1B**) revealed that the aP 2-dose schedule induced a predominantly IgG1 response, consistent with the known Th2-skewing effect of aP vaccines adjuvanted with aluminum salts. Both antenatal boosters also induced high levels of IgG1. However, for the Th1-related isotypes (IgG2a and IgG3), a critical divergence emerged: the aPPreg group showed negligible levels, similar to the aP 2-dose schedule, whereas the OMVPreg booster elicited a significant induction of both IgG2a and IgG3 (p < 0.001 compared to both aP 2-dose and aPPreg groups).

We next evaluated the response against pertussis toxin subunit A (PTxA), a single well-defined immunogenic subunit of pertussis toxin (**Figures 1D, E**). Total IgG levels (**Figure 1D**, left panel) showed that the aP 2-dose schedule exhibited significant antibody levels, as expected due to its composition (described in Materials and Methods section). PTxA-specific IgG levels were detected after the booster dose, but no significant difference was observed between the aPPreg and OMVPreg groups. The isotype analysis (**Figure 1E**) revealed that both boosters induced IgG1. However, in contrast to the response against HKBp, the OMVPreg group did not induce detectable levels of IgG2a or IgG3 against PTxA, and the aPPreg group showed only negligible levels (p = n.s. compared to aP 2-dose schedule). These findings should be interpreted with caution, as the commercial aP vaccine contains a substantially higher concentration of PTx than the OMV preparation (39)⁠. Thus, the absence of a clear Th1-associated isotype response against PTxA in the OMVPreg group may reflect the lower antigen dose rather than an intrinsic limitation of the OMV platform to induce Th1 responses against this antigen. Consistent with this interpretation, when the same OMVPreg vaccine was tested against the complex HKBp antigenic repertoire (which includes multiple bacterial components naturally present at higher or more diverse concentrations), a robust IgG2a/IgG3 response was readily observed (**Figure 1C**). Therefore, the ability of OMVPreg to induce a Th1-biased response appears to be antigen-dose dependent, at least for PTxA. Importantly, the comparison between the robust response against the complex HKBp antigenic repertoire and the comparatively limited response against the single PTxA antigen underscores that the OMV platform elicits a more comprehensive humoral response, recognizing a wider array of pathogen components beyond the dominant acellular vaccine antigens. Furthermore, these data demonstrate that the final immune signature induced by the antenatal booster is not merely a consequence of administering this dose but rather depends critically on the nature of the vaccine used. While a third dose of aP maintained the Th2-skewed profile established by the two priming doses, a third dose of OMVPreg fundamentally redirected the response toward a balanced Th1/Th2 antibody isotype signature, highlighting the distinct immunomodulatory properties of the OMV platform.

We also evaluated maternal antibody levels against the heterologous antigens tetanus and diphtheria toxoids, which are present in the commercial aP vaccine and included in the OMV formulation here tested (**Supplementary Figure 1**). Total IgG levels against tetanus toxoid showed that the aP 2-dose schedule induced baseline levels (**Supplementary Figure 1A**). Both antenatal boosters increased these IgG levels, without exhibiting significant difference between OMVPreg and aPPreg. The isotype profile against tetanus (**Supplementary Figure 1B**) revealed that OMVPreg induced a clear shift toward IgG2a and IgG3, while aPPreg and the aP 2-dose schedule remained IgG1-dominant. The same pattern was observed for diphtheria toxoid: the isotype profile (**Supplementary Figure 1D**) showed that only OMVPreg induced detectable IgG2a and IgG3. These findings are particularly noteworthy because they add a novel dimension to our understanding of the OMV platform. While it was already known that OMVs possess intrinsic adjuvant and Th1-modulatory properties (42)⁠, the present data demonstrate that a single OMV dose administered during gestation is sufficient to redirect the immune response toward a Th1- antibody isotype biased profile even in animals with a pre-existing Th2-skewed background established by two prior aP immunizations. This capacity to overcome an established Th2 memory and impose a balanced Th1/Th2 isotype signature response with a single booster has not been previously described and underscores the potency of the OMV platform as an immune modulator.

Collectively, these results demonstrate that while both vaccines effectively boost antibody levels, the OMV platform uniquely broadens the maternal immune signature. The induction of a balanced antibody profile, characterized by significant IgG2a and IgG3, is functionally relevant for pertussis-specific responses and, importantly, is also observed against heterologous vaccine antigens. These immunological advantages were achieved without compromising maternal or reproductive outcomes, as dams in all groups remained healthy and produced litters of comparable size with no pup mortality.

### Humoral immune response in the offspring

As described in Materials and Methods section, for all experiments involving offspring, litters rather than individual pups were used as the experimental unit.

**Figure 2:**
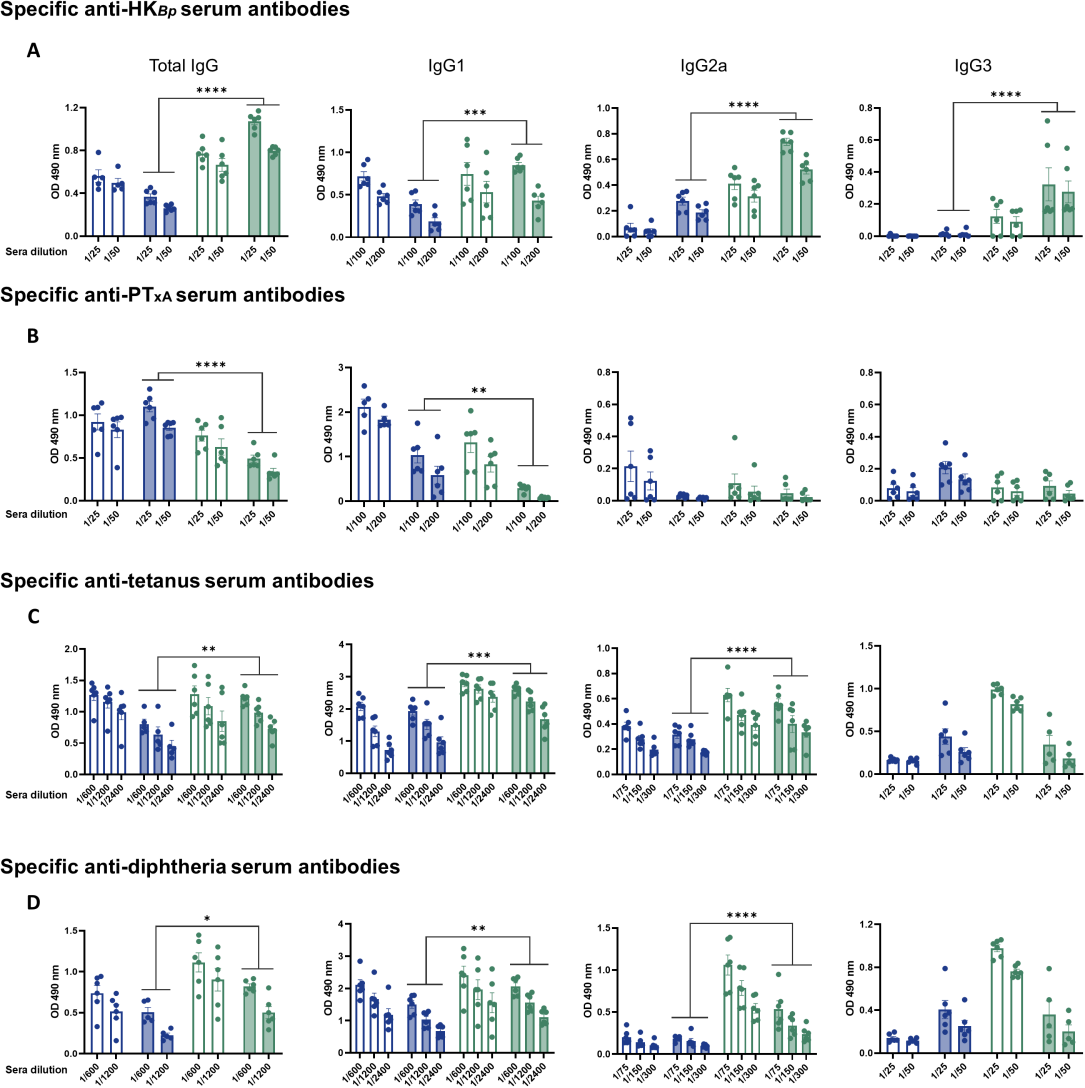
Humoral immune response induced by antenatal immunization in dams and their respective litters. Humoral immune response in dams was evaluated in serum collected 28 days after the antenatal booster, while antibody transfer to their offspring was assessed in serum samples collected at 21 days of age (infants).For offspring, each dot represents the mean value of a litter (rather than individual pups).Total IgG, IgG1, IgG2a and IgG3 levels specific to a heat-killed *B. pertussis* lysate (HKBp, **A**), pertussis toxin (PTxA, **B**), tetanus toxoid **(C)** and diphtheria toxoid **(D)** were measured by ELISA. Blue bars represent aPPreg treatment whereas green bars correspond to OMVPreg, empty bars display responses of dams and filled bars represent offspring. Optical density (OD) at 490 nm was determined for two or three serum dilutions. Statistical significance: *p<0.05, **p<0.01, ***p<0.001, ****p<0.0001 (two-way ANOVA, Sidak’s multiple comparisons)..

For humoral analysis serum samples were collected from each litter born to immunized mothers within 3–12 weeks after the antenatal boost. Comparisons were performed between litters from aP-vaccinated dams and those from OMV-vaccinated dams. Serum samples from litters born to non-immunized control dams were also collected. Antibody levels and isotype profiles against HKBp, PTxA, tetanus toxoid, and diphtheria toxoid were measured (**Figure 2**).

Total IgG levels against HKBp in the offspring are shown in **Figure 2A** (left panel). Pups born control dams exhibited non detectable antibody levels (not shown). Offspring of aPPreg-vaccinated dams showed significantly higher total IgG levels compared to control offspring, indicating efficient transfer of maternal antibodies induced by the antenatal aP booster. Litters from OMVPreg-vaccinated dams achieved the highest total IgG levels, significantly surpassing the aPPreg litters (p < 0.0001). The isotype profile against HKBp in the litters mirrored the pattern observed in their mothers. Litters from aPPreg dams exhibited a predominantly IgG1 response, with almost negligible IgG2a and IgG3. In contrast, litters from OMVPreg dams displayed a balanced isotype profile with significant levels of both IgG1 and the Th1-associated isotypes IgG2a and IgG3 (p < 0.0001 compared to aPPreg litters for IgG2a and IgG3). These results demonstrate that the qualitative shift toward a mixed Th1/Th2 antibody isotype signature induced by OMVPreg in the mothers is efficiently transferred to the progeny.

We next examined the response against PTxA in the litters (**Figure 2B**). Litters from aPPreg-vaccinated and OMVPreg-vaccinated dams both showed significantly elevated total IgG levels compared to control litters (p < 0.05 for both comparisons), with levels being higher in the aPPreg offspring in comparison with OMVPreg offspring groups (p < 0.0001). The isotype profile against PTxA revealed that both groups maintained an IgG1-dominant response, with negligible IgG2a and IgG3, and no significant differences were observed between aPPreg and OMVPreg litters for any isotype. These findings are consistent with the maternal data and reflect the lower PTx content in the OMV preparation, as discussed above. Nevertheless, the presence of total IgG anti-PTxA in aPPreg and OMVPreg litters confirm that both vaccines effectively transfer PTx-specific antibodies to the progeny.

We also evaluated the litters antibody response against the heterologous antigens tetanus and diphtheria toxoids (**Figures 2C, D**). Total IgG levels against tetanus (**Figure 2C**, left panel) showed that litters from OMVPreg-vaccinated dams exhibited significantly higher levels than litters from aPPreg-vaccinated dams (p < 0.01). Litters from aPPreg-vaccinated dams also showed higher levels than control litters (not shown). The isotype profile against tetanus revealed that litters from OMVPreg-vaccinated dams displayed higher IgG1 and IgG2a with significant differences compared to aPPreg litters (p < 0.001 for IgG1 and IgG2a). Litters from aPPreg-vaccinated dams remained IgG1-dominant, with low levels detected for IgG2a/IgG3.

The same pattern was observed for diphtheria toxoid (**Figure 2D**). Total IgG levels (left panel) were significantly higher in litters from OMVPreg-vaccinated dams compared to aPPreg litters (p < 0.05). The isotype profile showed that only OMVPreg litters exhibited a shift toward IgG2a, with significant differences compared to aPPreg litters (p < 0.0001). Litters from aPPreg-vaccinated dams showed low levels IgG2a/IgG3. These data demonstrate that the immunomodulatory effect of OMVPreg is not limited to pertussis-specific antigens but extends to heterologous vaccine components, and that this enhanced and balanced humoral response is successfully transferred to the next generation.

Levels of IgG and IgG isotypes were not detected in non-immunized mice (data not shown).

Taken together, these results indicate that the distinct maternal immune signatures induced by aPPreg and OMVPreg are efficiently passed to the offspring.

### Duration of humoral immunity in litters born at different times after maternal boosting

**Figure 3:**
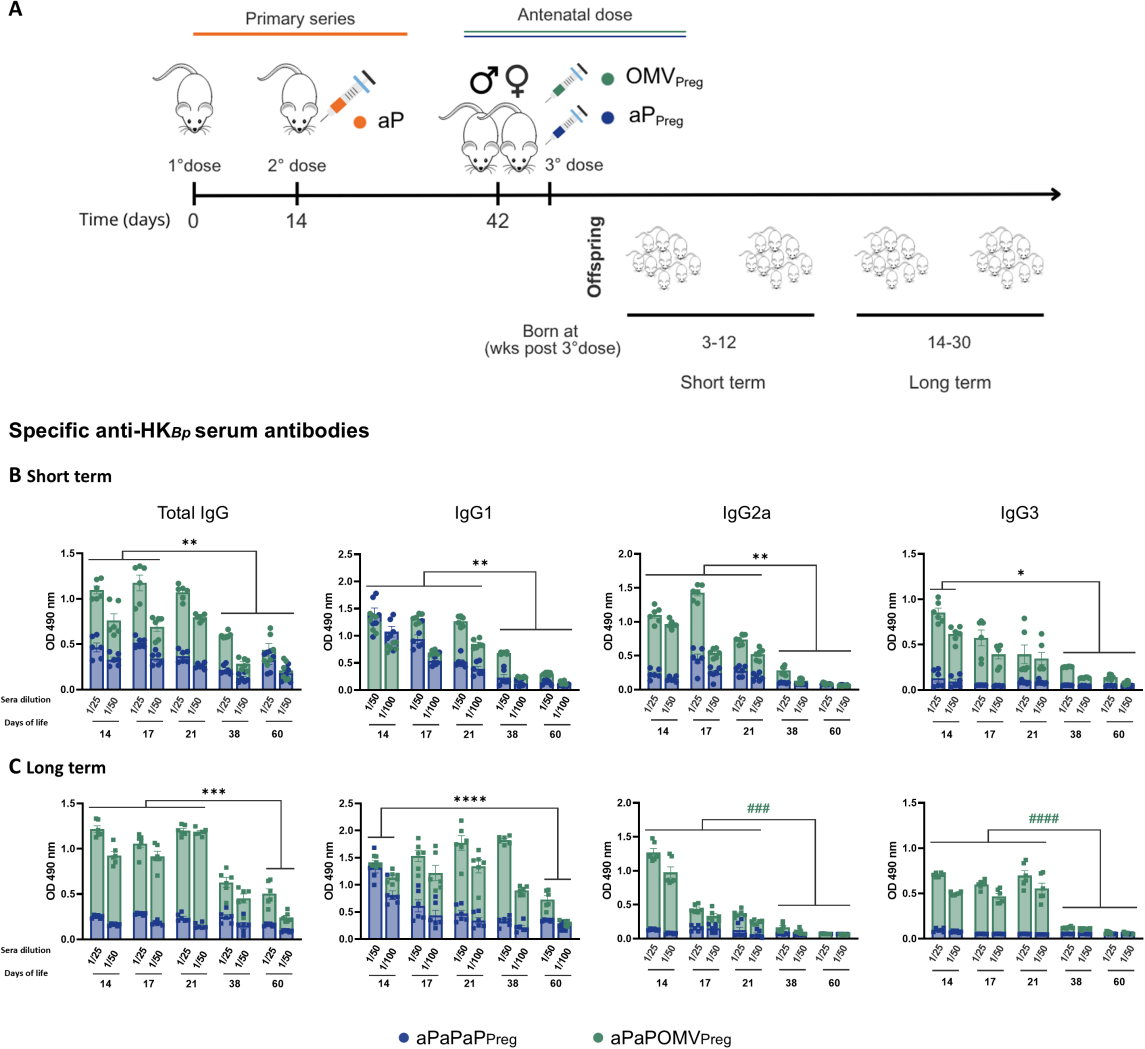
Humoral immune response detected in litters born at different intervals after the antenatal aPPreg or OMVPreg booster dose. Schematic representation of the experimental design is presented in panel **(A)** Offspring born to immunized dams at different timepoints after the antenatal booster were analyzed. Litters born between 3–12 weeks after the booster were defined as short-term litters, whereas those born between 14–30 weeks were defined as long-term litters. Each point represents the mean value of a litter. To assess the duration of maternal immunity in the offspring, serum samples of litters born at short **(B)** or long term **(C)** after the antenatal dose were collected at different ages. Total IgG, IgG1, IgG2a and IgG3 levels specific to HKBp were measured by ELISA. Optical density (OD) at 490 nm was determined for two serum dilutions. Each bar is split to display responses in offspring from aPPreg (blue) and OMVPreg (green) groups. Statistical significance: *p<0.05, **p<0.01, ***p<0.001, ****p<0.0001 (two-way ANOVA, Sidak’s multiple comparisons comparing column means). Statistical differences refer to the kinetics of antibodies levels rather than differences between both groups. Asterisks indicate differences observed in both treatments (aPPreg and OMVPreg). ### p<0.001, #### p<0.0001 indicate differences between kinetics observed only in the OMVPreg group..

**Figure 4:**
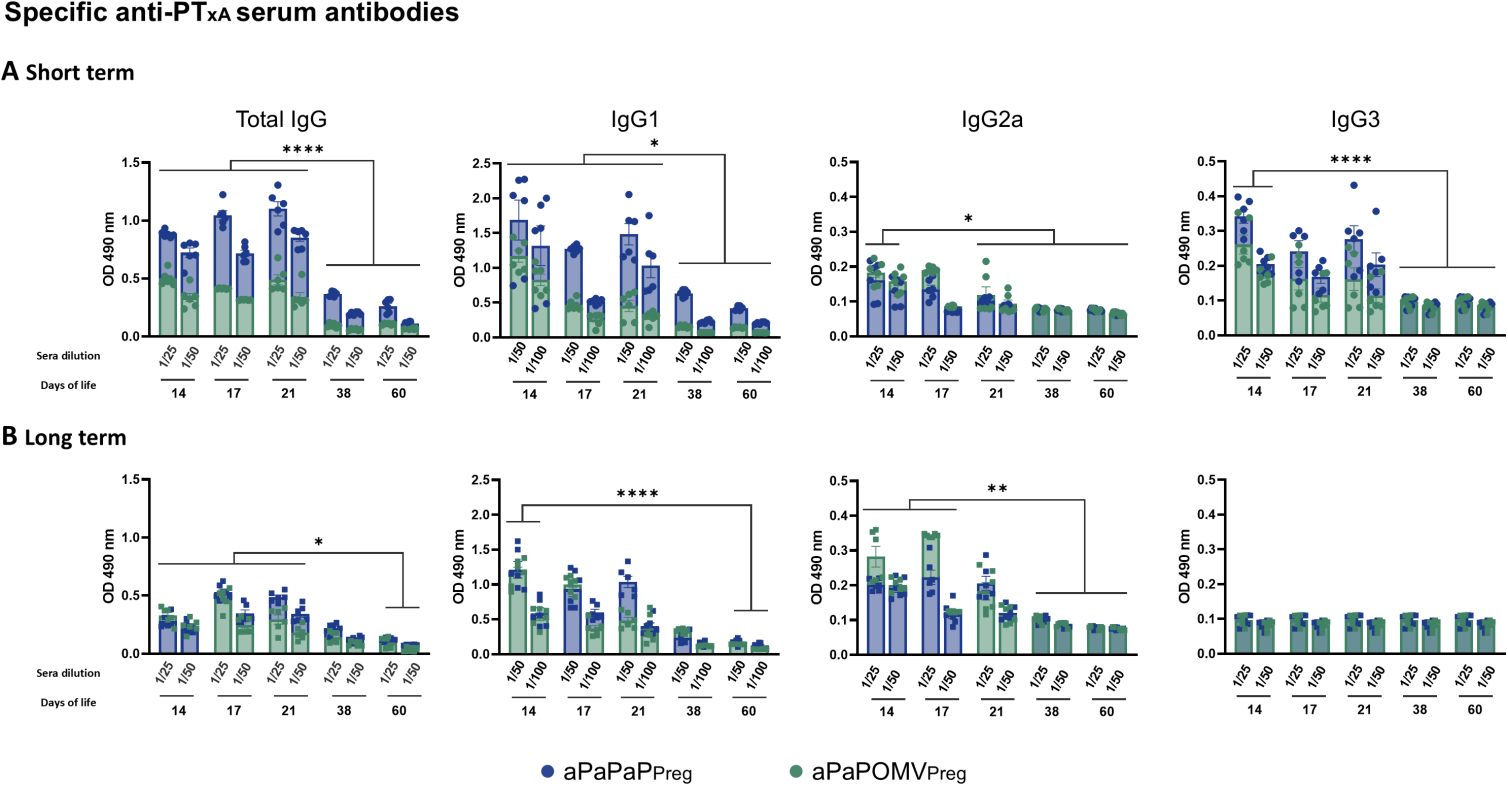
Humoral immune response specific to pertussis toxin induced in litters after maternal immune transfer of antibodies. To assess the duration of transferred maternal antibodies in offspring, serum samples of litters born at short **(A)** or long term **(B)** after the antenatal dose were collected at different ages. Total IgG, IgG1, IgG2a and IgG3 levels specific to pertussis toxin (PTxA) were measured by ELISA. Optical density (OD) at 490 nm was determined for two serum dilutions. Each point represents the mean value of a litter. Statistical significance: *p<0.05, **p<0.01, ****p<0.0001 (two-way ANOVA, Sidak’s multiple comparisons comparing column means)..

We next evaluated the duration of passively transferred immunity in litters born at different time intervals after the antenatal booster. The experimental design is outlined in **Figure 3A**. Litters were classified into two groups: those born shortly after the maternal boost (short term, within 3–12 weeks post-booster) and those born later (long term, within 14–30 weeks post-booster). Both groups were evaluated up to 60 days of age. The two maternal groups were here compared: aPPreg-vaccinated dams (blue symbols) and OMVPreg-vaccinated dams (green symbols). Non-immunized controls were uniformly non-detectable (data not shown). Antibody levels and isotype profiles against HKBp (**Figure 3**) and PTxA (**Figure 4**) were measured.

In litters born shortly after the antenatal boost (short term, **Figure 3B**), total IgG levels against HKBp were significantly higher in the OMVPreg group compared to the aPPreg group at all time points evaluated up to 38 days of age (p < 0.0001). When we analyzed the decline of antibodies over time, a marked difference emerged between the two groups. In aPPreg litters, total IgG levels against HKBp began to decrease as early as day 21 and fell substantially by day 38. In contrast, OMVPreg litters exhibited a slower decline: total IgG levels remained relatively stable from day 14 to day 21, and although they decreased by day 38, the levels were still significantly higher than those in aPPreg litters. The isotype profile against HKBp revealed even more striking differences. In aPPreg litters, the IgG1 response peaked at day 14 and declined rapidly thereafter, becoming barely detectable by day 60. IgG2a and IgG3 were virtually absent at all time points. In contrast, OMVPreg litters not only exhibited higher peak levels of IgG1, IgG2a, and IgG3 at day 14, but also showed a significantly slower decline for all three isotypes. IgG1 remained detectable at higher levels throughout the 38 day period compared to aPPreg litters. Notably, IgG2a and IgG3, which were barely detected in aPPreg litters, declined slowly in OMVPreg litters and remained detectable up to day 38 (p < 0.05 compared to aPPreg litters for IgG2a and IgG3 at all time points).

In litters born later after the maternal boost (long term, more than 14 weeks post-booster, **Figure 3C**), total IgG levels against HKBp were significantly lower in aPPreg group compared to short-term litters, reflecting the longer interval since maternal immunization. However, OMVPreg litters still exhibited significantly higher total IgG levels, with slower decline starting at 38 days of age, than aPPreg litters at all time points evaluated up to 60 days of age (p < 0.0001). aPPreg litters showed low IgG levels up to the tested day 60. Analysis of the isotype profile revealed distinct kinetics for each isotype. For aPPreg litters, IgG1 declined rapidly, beginning as early as day 17 and continuing to decrease thereafter, with no detectable IgG2a or IgG3 at any time point. In contrast, OMVPreg litters exhibited a more sustained profile. IgG1 remained stable until approximately day 38 and only began to decline by day 60. IgG2a showed significant levels at day 14 but declined thereafter. Notably, IgG3 sustained its levels until at least day 21 of age, with a subsequent decline observed by day 38. These results demonstrate that OMVPreg litters maintain a more durable and balanced isotype profile, with prolonged persistence of IgG1 and IgG3, and an early but transient peak of IgG2a.

Total specific PTxA IgG levels and isotype profiles were also evaluated in litters born shortly after the antenatal boost (short term, **Figure 4A**) and in those born later (long term, post booster, **Figure 4B**). Both groups were evaluated at the same time points of age up to 60 days.

In short term litters (**Figure 4A**), total IgG levels against PTxA showed significant difference between aPPreg and OMVPreg groups at any time point evaluated (p < 0.05). The isotype profile in aPPreg litters was exclusively IgG1 dominant, with barely low levels of IgG2a and IgG3 at all time points. OMVPreg litters exhibited a similar behavior as aPPreg litters as expected due its PTx content. In the long term litters (**Figure 4B**), however, OMVPreg exhibited higher levels of IgG2a at 14 and 17 days of age in comparison to aP offspring of the same age. Notably, IgG and IgG1 levels were sustained over the long term in OMVPreg litters, whereas these antibodies were diminished in aPPreg litters.

We also evaluated the humoral immune response in the mothers themselves at the same time points corresponding to short term and long-term litters (**Supplementary Figure 2**). In mothers vaccinated with aPPreg, total IgG and isotype levels against both HKBp and PTxA either remained sustained or declined significantly between short term and long-term time points, with the exception of IgG2a, which showed a slight increase. In contrast, mothers vaccinated with OMVPreg maintained or significantly increased total IgG levels and all isotypes against HKBp between short term and long-term time points (p < 0.05). Against PTxA, OMVPreg mothers behaved similarly to aPPreg mothers (p < 0.05). These maternal data provide a direct correlate for the enhanced and more durable passive immunity observed in the progeny of OMVPreg vaccinated dams.

### Protective efficacy against *B. pertussis* challenge in offspring of different ages

**Figure 5:**
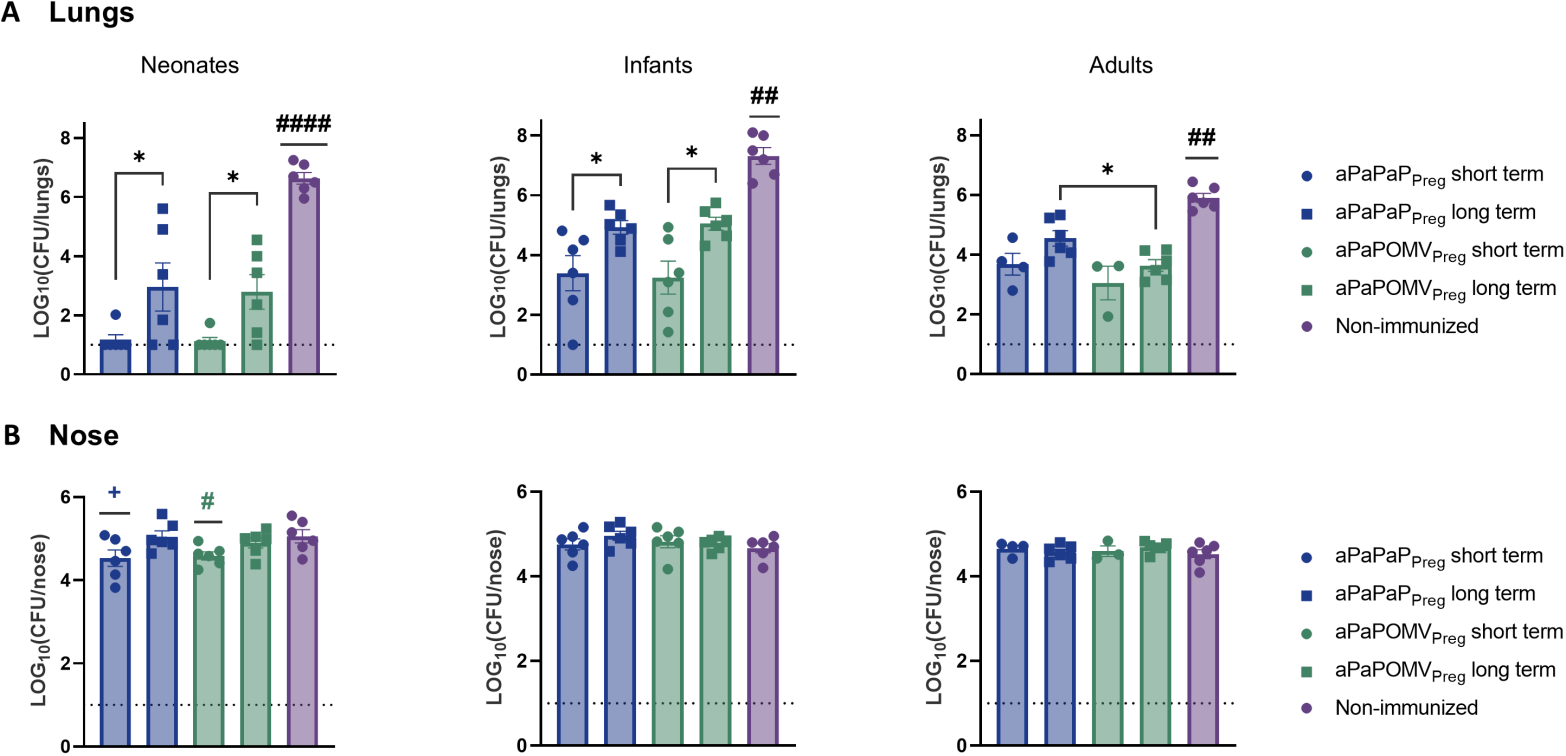
Protective capacity against *B. pertussis* in litters of different ages.​ The protective capacity of maternally transferred immunity was evaluated in offspring of different ages following intranasal challenge with *B. pertussis*. Sublethal doses were as follows: 5×10^4^ CFU for neonates, and 5×10^6^ CFU for infants and adults. Bacterial burden was assessed 7 days post-challenge in the lungs **(A)** and nose **(B)**. Results are expressed as log_10_ CFU per organ (mean ± SEM). Blue bars represent aPPreg, green bars correspond to OMVPreg, and purple bars represent offspring born to non-immunized dams. Circles indicate litters born at short term after antenatal dose, whereas squares indicate long term litters. Each point represents the mean value of a litter. Statistical analysis was performed using one-way ANOVA, followed by Tukey’s for multiple comparisons. *p<0.05 corresponds to a statistical difference between treatments shown with brackets. ##p<0.01, ####p<0.0001 indicate differences between one treatment and all the others. In **(B)**, **+** and # indicate *p<0.05 for comparison between the short- and long-term groups within the same treatments, as well as against the non-immunized control..

To evaluate the protective breadth of antenatal immunization, we quantified bacterial clearance in the respiratory tract of litters from aPPreg vaccinated and OMVPreg vaccinated dams, compared to non-immunized controls (**Figure 5**). Protection was assessed by bacterial burden (log_10_ CFU) recovered from lungs (primary readout for severe disease) and nose (indicator of transmission potential) 7 days after challenge. This time point captures the peak of infection before bacterial clearance becomes dominant, providing a clearer measure of the vaccine’s capacity to control bacterial replication. Litters were challenged at three developmental stages: neonates (7 days old, sublethal dose 5×10^4^ CFU of *B. pertussis*), infants (21 days old, challenge dose 5×10^6^ CFU), and adults (60 days old, challenge dose 5 × 10^6^ CFU). For each age, litters born shortly after the antenatal boost (short term) and those born later (long term) were evaluated separately.

In neonatal litters, both aPPreg and OMVPreg induced robust pulmonary protection in short term born litters (bars with circles). Bacterial loads in the lungs were reduced from approximately 6.6 log_10_ CFU in non-immunized controls to less than 1.5 log_10_ CFU in both vaccine groups (p < 0.0001 for each vaccine group vs control), with no significant differences between aPPreg and OMVPreg litters. Nasal bacterial loads followed a similar pattern. In short-term litters, both vaccine groups exhibited significantly lower nasal colonization relative to non-immunized controls (p < 0.01). Notably, for both aPPreg and OMVPreg, nasal protection waned significantly in long-term litters compared to short-term litters (p < 0.05), indicating a loss of efficacy over time. No significant differences were observed between aPPreg and OMVPreg litters within either period. In long term born neonatal litters (bars with squares), a gradual decline in protection was detected in lungs, but litters from both vaccine groups still maintained a significant reduction in bacterial burden (at least 3.5 log_10_ CFU lower than controls, p < 0.0001), with no differences between aPPreg and OMVPreg offspring.

In infant offspring (21 days old) from short term litters, both aPPreg and OMVPreg continued to provide strong protection against the challenge dose of 5 × 10^6^ CFU. In the lungs, OMVPreg offspring showed a reduction of over 4 log_10_ CFU compared to non-immunized controls (p < 0.0001), and aPPreg litters showed a similar reduction (p < 0.001 compared to controls). No significant differences in pulmonary bacterial burden were observed between the two vaccine groups. Nasal colonization was not reduced in either vaccine groups compared to controls. In long term born infant litters, protection was reduced in both immune groups with a reduction of over 2 log_10_ CFU compared to non-immunized controls (p < 0.01), with no significant differences between aPPreg and OMVPreg litters.

In adult offspring (60 days old) from short-term litters, both vaccines provided protection against the challenge dose of 5 × 10^6^ CFU (p < 0.01 *vs* non-immunized control), with no significant differences between both immune litters. However, in adult offspring born from long term litters, a striking difference emerged between the two vaccine groups. In the lungs, aPPreg litters showed a relative loss of protective efficacy, with pulmonary bacterial counts significantly higher (> 4.5 log_10_ CFU) compared to OMVPreg litters (< 4 log_10_ CFU; p < 0.01). Both vaccine groups still showed significant reductions compared to non- immunized controls (p < 0.01). In the nose, no reduction was observed for any of the treatments studied here. These results demonstrate that while both vaccines protect neonates and infants equivalently regardless of short-term or long-term birth window, OMVPreg provides superior and more durable protection in adult offspring born from late litters, particularly in the lungs.

### Cellular immune response in the offspring upon *B. pertussis* encounter

**Figure 6:**
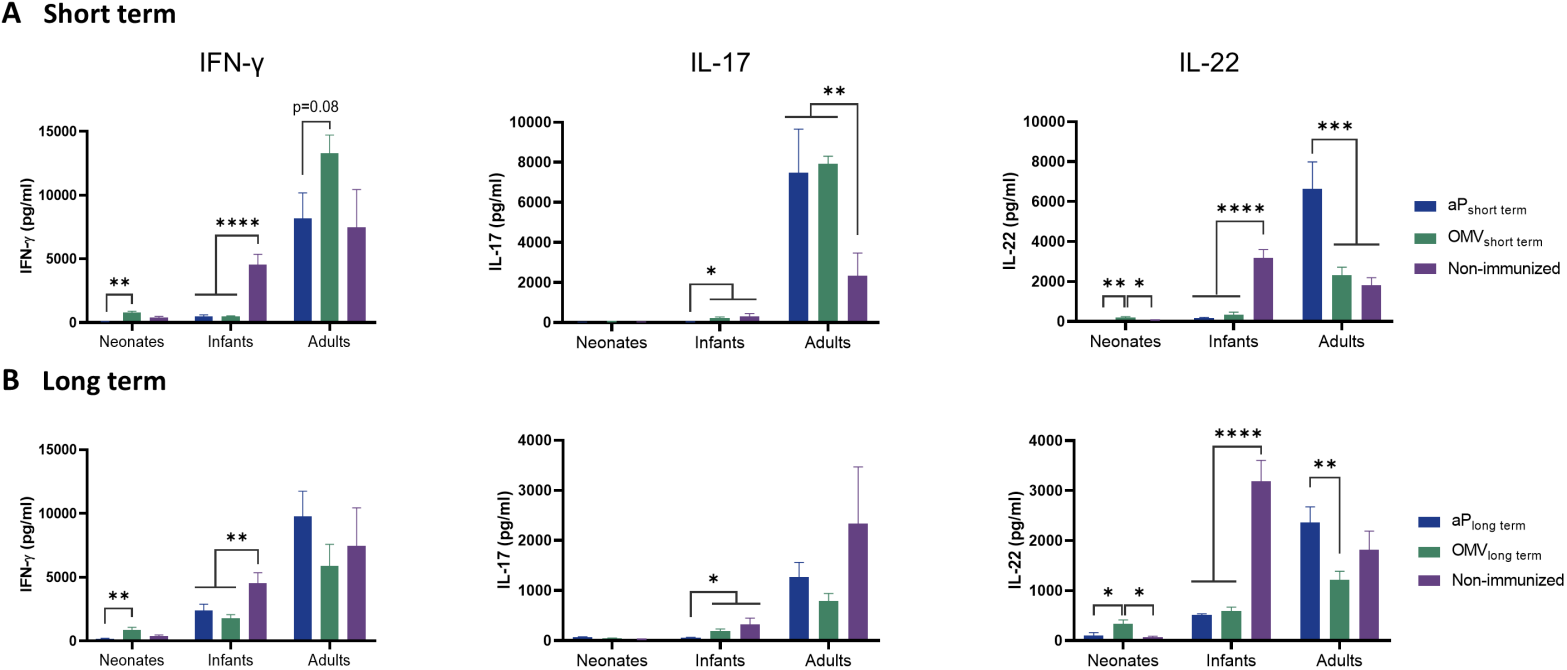
Cellular immune response induced in litters after antenatal immune transfer of antibodies. To evaluate the cellular immune response that offspring were able to mount upon encountering the pathogen, we measured cytokine production in spleen cells collected after ex vivo stimulation with a *B. pertussis* whole lysate. A non-stimulated control was included in all experiments (data not shown; cytokine levels were undetectable). After 72 hours of incubation, concentrations of IFN-γ, IL-17, and IL-22 in culture supernatants were measured by ELISA. This cellular response was assessed in offspring of all ages (neonates, infants, and adults). Blue column represent aPPreg litters, green column represent OMVPreg litters, and purple column represent non-immunized infected controls. Data represents the mean value of the litters ± SEM. Cytokine levels were evaluated in offspring born in early litters (short term, **A**) and late litters (long term, **B**). Statistical analysis was performed using one-way ANOVA followed by Tukey’s test for multiple comparisons. *p < 0.05, **p < 0.01, ***p < 0.001, and ***p < 0.0001 indicate differences between one treatment and all others.

To evaluate the cellular immune response that litters were able to mount upon encountering the pathogen we measured cytokine production in spleen cells collected after *ex vivo s*timulation with a *B. pertussis* whole lysate (**Figure 6**). This was evaluated in litters born in the short term and those born in the long term after the antenatal booster. Responses were compared between litters from aPPreg vaccinated dams, litters from OMVPreg vaccinated dams, and control litters that received no maternal immunity and were infected (infected alone).

In litters of neonates, the levels of IFN-γ, IL-17, or IL-22 were low in all groups tested here compared to other ages, regardless of antenatal immunization status or term of birth, consistent with the immunological immaturity at this age.

In litters of infants, both aPPreg and OMVPreg litters exhibited lower levels of IFN-γ (p < 0.01) and IL-22 (p < 0.0001) compared to infected only control litters, regardless of being born in the short or long term after the antenatal booster. This is likely explained by the lower bacterial colonization in these groups due to the protective effect of transferred maternal immunity; with less pathogen burden, the induced cellular response is consequently lower. No significant differences in cytokine levels were observed between aPPreg and OMVPreg infants with the exception of IL-17 that was higher in OMVPreg in comparison to aPPreg.

In adult litters, a more complex pattern emerged. In short term adults, both aPPreg and OMVPreg offspring exhibited detectable levels of IFN-γ, IL-17, and IL-22. IFN-γ levels were higher in OMVPreg litters compared to aPPreg offspring. IL-17 levels were similar between both vaccine groups. IL-22 levels were higher in aPPreg litters compared to OMVPreg offspring (p < 0.001). In long term adults, aPPreg litters showed levels of IFN-γ, IL-17, and IL-22 that were not significantly different from infected only control litters, indicating that the cellular recall response in aPPreg adults is largely driven by the infection itself rather than by transferred maternal immunity, consistent with the higher bacterial colonization observed in this group. In contrast, OMVPreg long term adults maintained lower IL-22 levels compared to infected only controls, correlating with their lower colonization, while IFN-γ and IL-17 remained detectable but at levels that reflect both the residual maternal immunity and the infection burden. These results demonstrate that maternal immunization with OMVPreg, unlike aPPreg, promotes a cellular response phenotype in offspring upon pathogen encounter (even when colonization levels are low) that is characterized by IFN-γ and IL-22 production.

## Discussion

The enduring resurgence of pertussis despite high vaccination coverage has exposed fundamental flaws in current acellular vaccines. They prevent disease but do not efficiently prevent nasal colonization or transmission (46–48)⁠⁠ and the immunity they induce is short lived due to their predominantly Th2 skewed response and failure to generate tissue resident memory CD4+ T cells (28, 49)⁠. Undoubtedly, aP vaccines remain a valuable tool that should be used while new vaccine developments progress (30, 32)⁠. In fact, their use during pregnancy has proven to be a clearly safe and effective strategy to reduce morbidity and mortality in the most vulnerable population group: newborns and infants until they receive their first infant dose (10–14, 50). However, the global shift to acellular vaccines has raised concerns regarding the duration of transferred immunity, largely due to the Th2 skewed profile they induce. In this study, we evaluated whether a single dose of a prototype *B. pertussis* outer membrane vesicle (OMV) vaccine, administered during pregnancy, could overcome these constraints. This candidate induces a more balanced immune profile (39)⁠ and tissue resident memory responses (36, 49)⁠, and we asked whether antenatal immunization with this prototype could transfer this profile to the offspring, thereby providing more durable and qualitatively superior protection.

Experiments conducted using the pregnancy model already established in our laboratory showed that despite the Th2 biased memory induced by two prior aP doses and the Th2 skewed environment of gestation, a single OMVPreg booster elicited a qualitatively different isotype profile, shifting the response toward a mixed IgG pattern (**Figure 1**). In fact, the OMVPreg induced a mixed IgG1/IgG2a/IgG3 isotype profile against complex *B. pertussis* antigens and, notably, also against heterologous vaccine components (tetanus and diphtheria toxoids, **Supplementary Figure 1**). This is remarkable because a third aP dose formulated with aluminum salts merely reinforced the existing Th2 profile (**Figure 1**). Thus, the OMV platform not only acts as a potent immunogen but also as an immune modulator capable of redirecting an established Th2 memory. This is a relevant result, as it demonstrates that an antenatal vaccine can overcome pre-existing Th2 skewing induced by 2 aP doses-primary series and induce a balanced immune profile in the mother, a finding with broad implications for antenatal immunization beyond pertussis.

This balanced maternal immunity was faithfully transferred to the litters (**Figure 2**). Litters born to OMVPreg vaccinated dams received not only higher total IgG levels but also functionally relevant Th1 associated isotypes (IgG2a and IgG3), which were almost absent in aPPreg litters (**Figure 2**). Moreover, these qualitative differences translated into distinct protective outcomes. While both vaccines protected neonates and infants born shortly after the booster, a striking divergence emerged in adult offspring from late litters. Here, aPPreg litters had lost protective efficacy, with pulmonary bacterial loads approaching those of non-immunized controls (a reduction of only 1.35 log_10_ CFU *vs* control group, p < 0.001). In contrast, OMVPreg litters maintained more robust bacterial clearance (a reduction of 2.3 log_10_ CFU, p < 0.001 *vs* control group).

The lower anti PTxA IgG2a/IgG3 response in OMVPreg litters reflects the lower PTx content in our OMV preparation compared to the commercial aP vaccine. However, this did not compromise overall protective efficacy, as OMVPreg litters were equally or better protected than aPPreg offspring against bacterial challenge. This observation reinforces the notion that protection against pertussis is multifactorial and does not rely solely on anti PTx antibodies. Indeed, the robust response against the complex HKBp antigenic repertoire indicates that OMVs induce broad, non-redundant humoral immunity that recognizes multiple bacterial components, a feature likely critical for long term protection.

A novel finding of this study is that the advantage of OMVPreg extends beyond passive antibody transfer. In infant offspring, despite having significant bacterial colonization, albeit lower than that of infected only controls, the levels of all three cytokines (IFN-γ, IL-17, IL-22) were lower than those observed in infected only controls. This indicates that maternal immunity does not simply dampen the offspring’s own response but can prime it for a more robust recall, while the lower cytokine levels primarily reflect the reduced pathogen burden due to effective protection.

Notably, in adults, aPPreg litters showed higher IL22 levels compared to OMVPreg litters. This skewing toward IL 22 in the aP group may be related to the presence of maternal microchimerism (51–53), which could influence the litter’s immune phenotype in a vaccine specific manner.

In long-term litters of adults, the superiority of OMVPreg became evident. Litters of OMVPreg vaccinated dams maintained significantly lower bacterial colonization compared to aPPreg litters. As a consequence, their IL-17 and IL-22 levels were lower, reflecting the reduced pathogen burden. Importantly, the difference in IL-22 levels between OMVPreg and aPPreg long term adults was statistically significant (p < 0.01). This indicates that OMVPreg mediated protection is not only more effective but also avoids the higher IL-22 response seen in aPPreg litters, which may be associated with higher inflammation. In summary, these findings demonstrate that OMVPreg, unlike aPPreg, elicits an immune response that conditions offspring of the litters to mount a balanced and proportionate cytokine response upon infection, rather than an exacerbated response driven primarily by pathogen burden.

In litters of neonates, encounter with the pathogen did not induce a significant cellular response in any group in comparison with other ages studied here. This is consistent with the immunological immaturity of this age, where regulatory mechanisms predominate and the capacity to mount Th1/Th17 type responses is still underdeveloped. Therefore, early life protection relies almost entirely on passively transferred antibodies rather than on cellular immunity.

Attenuated BPZE1 and antibiotic inactivated (AIBP) prototype vaccines have also shown great promise in inducing Th1/Th17 immunity and preventing nasal colonization, but they require intranasal administration. An attenuated vaccine cannot be used in pregnant women. While intranasal delivery may be optimal for inducing respiratory TRM cells, it poses practical and regulatory challenges for routine use, particularly in pregnant women. Moreover, AIBP prototype vaccine should not be accepted based on adverse reaction associated to wP vaccine (48). Our OMVPreg vaccine is administered intramuscularly, the same route already established for antenatal immunization programs, yet it still conferred a balanced and durable immune profile to the offspring. This practical advantage, combined with its ability to redirect pre-existing Th2 immunity and to enhance responses to heterologous antigens, positions OMVPreg as a strong candidate for improving current antenatal pertussis vaccination strategies.

In our experimental setting, where protection in offspring relies on maternally transferred immunity following a single intramuscular OMV booster dose administered to dams (after two aP priming doses), achieving robust nasal protection may represent a more demanding goal. This is attributable to the fact that passive antibody transfer constitutes the primary mechanism of protection in neonates and infants, and systemically derived antibodies may be less effective at mucosal surfaces than locally induced immunity. Of note, although nasal protection was not consistently observed, a trend toward reduced nasal colonization was detected with lower challenge doses (preliminary data not shown), suggesting that the OMV platform retains the potential to confer nasal protection under optimized conditions.

Several limitations should be acknowledged. The murine model, while informative, does not fully recapitulate human pregnancy and infant immune development. Additionally, the lower PTx content in our OMV preparation limited the Th1-associated isotype response against this specific antigen, although overall protection was not compromised. It is also important to note that maternal T-cell transfer was not assessed in this study, and therefore our data demonstrate correlation between maternal isotype profile and litters responses, rather than causation. Finally, whether OMVPreg induces respiratory memory cells in the litters remains to be determined. Nonetheless, our findings already show a clear advantage in terms of durability and quality of protection.

In conclusion, unadjuvanted OMVs represent a promising next-generation platform for antenatal pertussis immunization, which could hypothetically reduce the need for vaccination during every pregnancy. By redirecting maternal immunity toward a balanced isotype and cellular profile, OMVPreg conferred durable and qualitatively superior protection to offspring, potentially overcoming key limitations of current aP-based strategies. The extension of these benefits to heterologous antigens and the ability to redirect pre-existing Th2 immunity position OMV as a versatile and potent candidate for improving antenatal and neonatal health. Nevertheless, translation of these findings to humans must consider key differences in IgG transfer between mice and humans, including isotype-dependent placental transfer efficiency (54), as well as the potential contribution of IgA to mucosal protection. Future studies in relevant models will be essential to address these considerations.

## Data Availability

The datasets presented in this study can be found in online repositories. The names of the repository/repositories and accession number(s) can be found in the article/**Supplementary Material**.

## Supplementary material

Supplementary Figure S1 Humoral immune response specific to tetanus and diphtheria toxoids induced by maternal immunization in a murine pregnancy model. Humoral immune responses to tetanus and diphtheria toxoids were evaluated by ELISA in serum samples collected 28 days after the primary series **(A, C)** and after the antenatal booster **(B, D)**. Total IgG, IgG1, IgG2a, and IgG3 levels specific to tetanus toxoid **(A, B)** and diphtheria toxoid **(C, D)** were measured. Optical density (OD) at 490 nm was determined for two or three dilutions. Sera from immunized mice were compared to non‑immunized controls (data not shown; undetectable). Statistical significance: **p < 0.01, ***p < 0.001, ***p < 0.0001 (two‑way ANOVA with Šídák’s multiple comparisons test).

Supplementary Figure S2 Kinetics of the humoral immune response to *B. pertussis* antigens induced at different timepoints after the antenatal dose. *B. pertussis*-specific antibody kinetic in dams was evaluated by ELISA in serum collected at short term (circles) or long term (squares) after the antenatal booster. Total IgG, IgG1, IgG2a, and IgG3 against a heat‑killed B. pertussis lysate **(A)** and pertussis toxin **(B)** were measured. OD at 490 nm was determined for two serum dilutions. Significance across all dilutions was determined by two‑way ANOVA with Šídák’s. *p < 0.05, **p < 0.01, ***p < 0.001, ***p < 0.0001.
